# Pituitary Imaging: Indications and Outcomes From a Tertiary Pediatric Center in the United Arab Emirates

**DOI:** 10.7759/cureus.99675

**Published:** 2025-12-19

**Authors:** Mohamad Sabsabee, Reham Ghanim, Ajay Prashanth, Mohammad El Abiary, Nandu Thalange

**Affiliations:** 1 Pediatrics, Al Jalila Children's Speciality Hospital, Dubai, ARE; 2 Pediatric Endocrinology, Glucare Integrated Diabetes and Endocrinology Center, Dubai, ARE; 3 Diagnostic Radiology, Al Jalila Children's Speciality Hospital, Dubai, ARE; 4 Pediatric Endocrinology, Al Jalila Children's Speciality Hospital, Dubai, ARE; 5 Pediatric Endocrinology, Genesis Healthcare, Dubai, ARE

**Keywords:** growth hormone deficiency, pituitary gland, pituitary mri, precocious puberty (pp), short stature (ss)

## Abstract

Introduction: The pituitary gland plays a critical role in regulating endocrine function, with disorders manifesting as diverse clinical and laboratory abnormalities. Magnetic resonance imaging (MRI) of the Sella turcica provides a detailed anatomical evaluation, supporting the diagnosis of pituitary pathologies, particularly growth hormone deficiency (GHD) and precocious puberty (PP).

Objectives: To describe the clinical, laboratory, and MRI characteristics of pediatric patients undergoing pituitary MRI at a tertiary pediatric center in the United Arab Emirates, and to estimate the diagnostic yield of MRI, defined as the frequency and pattern of structural pituitary abnormalities, across major endocrine indications.

Methodology: We conducted a retrospective review of 205 pediatric patients who underwent MRI Sella at Al Jalila Children’s Hospital, a major tertiary/quaternary pediatric hospital in Dubai, UAE, between January 2023 and June 2024. Data collected included demographics, auxological measurements (height, weight, BMI, bone age), laboratory results, and imaging findings.

Results: The cohort comprised 205 patients (130 (63.4%) male, mean age 10.0 years). Suspected GHD was the most common indication for MRI (165 patients, 80%), with 116 (56.5%) later confirmed. Pituitary hypoplasia was observed in 63 (54%) patients with GHD, while pituitary hyperplasia was noted in 5 (30%) cases of PP. Lesions such as Rathke’s cleft cysts (9, 4.4%) and interrupted pituitary stalk (5, 2.4%) were identified, predominantly in patients with GHD.

Conclusions: MRI sella provides anatomical characterization of the pituitary gland in children undergoing endocrine evaluation and demonstrates a range of structural findings, particularly among those with confirmed GHD. A comprehensive diagnostic approach combining imaging, auxological, and biochemical data enhances diagnostic accuracy.

## Introduction

The pituitary gland, seated within the sella turcica, is a central component of the hypothalamic-pituitary axis and regulates multiple endocrine functions essential for growth, metabolism, and reproduction. It comprises the anterior (adenohypophysis) and posterior (neurohypophysis) lobes, which secrete hormones including growth hormone (GH), thyroid-stimulating hormone (TSH), adrenocorticotropic hormone (ACTH), prolactin, and vasopressin [1,2]. Normal pituitary size and morphology vary across childhood and adolescence, with physiological enlargement occurring during puberty [3,4]. Structural or functional disturbances of the pituitary gland can result in diverse clinical manifestations, including growth disorders, pubertal abnormalities, and multiple pituitary hormone deficiencies.

Magnetic resonance imaging (MRI) of the sella turcica provides superior anatomical visualization of the pituitary gland, stalk, and surrounding structures, enabling assessment of pituitary volume, morphology, and focal lesions such as microadenomas, Rathke’s cleft cysts, hypoplasia, and pituitary stalk interruption syndrome [5-7]. These imaging findings complement auxological and biochemical evaluations and help distinguish pathological structural abnormalities from normal age-related variation.

Growth hormone deficiency (GHD) and central precocious puberty (CPP) are among the most common endocrine indications for pituitary MRI in children. GHD typically presents with short stature, poor height velocity, and delayed bone age, and can be associated with structural anomalies including pituitary hypoplasia, ectopic posterior pituitary, and interrupted stalk [8-10]. CPP results from premature activation of the hypothalamic-pituitary-gonadal axis, and MRI is primarily used to exclude hypothalamic or sellar lesions, particularly in younger children, boys, or those with atypical features [11-13].

International data suggest substantial variability in MRI diagnostic yield, with structural pituitary abnormalities identified in 40-60% of children with confirmed GHD [8-10,14-16], while the yield in CPP is considerably lower, especially in asymptomatic older girls [12,13,17]. Despite this, regional evidence from the Gulf and the Middle East is limited. Given differences in referral patterns, clinical practice, and population characteristics, understanding local imaging outcomes is essential.

This study was designed to address the lack of regional data on pituitary MRI utilization and findings in pediatric endocrine practice in the United Arab Emirates. The primary objective was to characterize the spectrum and frequency of MRI Sella findings in children undergoing pituitary imaging at a tertiary pediatric center, with particular focus on the prevalence of structural pituitary abnormalities in those with confirmed growth hormone deficiency. The secondary objectives were to describe the clinical indications prompting MRI referral, compare imaging findings across major diagnostic categories (including growth hormone deficiency and CPP), and estimate the diagnostic yield of MRI, defined as the proportion of studies demonstrating structural pituitary abnormalities. By describing current imaging patterns and correlating radiological findings with clinical and biochemical diagnoses, this study aims to provide locally relevant data to inform clinical decision-making and contextualize regional practice within existing international literature.

## Materials and methods

This retrospective, descriptive, observational single-center study was conducted at Al Jalila Children’s Specialty Hospital, a tertiary/quaternary pediatric referral center in Dubai, UAE. The study was approved by the Mohammed Bin Rashid University Institutional Review Board (Approval No. MBRU IRB-2024-118). Patient data were anonymized and de-identified before analysis, and no identifiable information was stored. The study was conducted in accordance with institutional ethical standards and the Declaration of Helsinki.

Study population

We included all children (<18 years) who underwent MRI Sella over a period of 18 months. Inclusion criteria: all pediatric patients who underwent MRI Sella for any endocrine, growth, or pituitary-related clinical indication during the study period.

Exclusion criteria: patients with (1) incomplete or technically inadequate MRI studies, (2) prior pituitary surgery or radiotherapy, or (3) missing key demographic or laboratory data. After applying these criteria, no cases met the exclusion criteria, and all 205 eligible patients were included in the final analysis.

Diagnostic definitions

GHD was defined as a peak growth hormone level <10 ng/mL on a provocative stimulation test in the presence of clinical and auxological features suggestive of GHD, in accordance with international pediatric endocrine guidelines. CPP was defined as the onset of secondary sexual characteristics before eight years in girls and nine years in boys, supported by clinical progression and biochemical evidence of central hypothalamic-pituitary-gonadal axis activation.

Pituitary hypoplasia was defined as a pituitary volume *z*-score < −2.0 for age and sex, calculated using established pediatric reference data.

Growth hormone stimulation testing was performed using oral clonidine at a dose of 0.15 mg/m² (maximum 0.25 mg). Blood samples for GH measurement were obtained at baseline and at 30, 60, 90, and 120 minutes post-administration. Blood pressure and heart rate were monitored throughout the procedure. A peak GH level below 10 ng/mL was considered an inadequate response.

Data collection

Data were extracted from the hospital’s electronic medical record system (Epic Systems Corp., Verona, WI) through structured chart review. The following parameters were collected:

(1) Demographic and auxological variables: age, sex, Tanner stage, height, weight, body mass index (BMI), bone age, and mid-parental height (expressed as *z*-scores)

(2) Laboratory data: GH stimulation test results, insulin-like growth factor-1 (IGF-1), and IGF-binding protein-3 (IGFBP-3) levels

(3) Radiological findings: pituitary volume, morphology, and focal lesions (hypoplasia, hyperplasia, Rathke’s cleft cysts, interrupted stalk, adenoma, or other anomalies)

MRI protocol and image analysis

All MRI studies were performed on a 3.0-Tesla Siemens MAGNETOM Skyra scanner (Siemens Healthineers, Erlangen, Germany) using a dedicated head coil.

The standard pituitary protocol included: sagittal and coronal T1-weighted sequences before and after intravenous gadolinium contrast, coronal T2-weighted sequences, and dynamic post-contrast T1 sequences for microadenoma detection.

Slice thickness was 2-3 mm. Pituitary height, width, and length were measured on midline sagittal and coronal planes.

Pituitary volume was calculated using the formula (length × height × width × 0.5) and converted to age- and sex-adjusted *z*-scores using published pediatric reference data [4]. 

Statistical analysis

Data were analyzed using IBM SPSS Statistics, version 30.0 (IBM Corp., Armonk, NY). Continuous variables were tested for normality using the Shapiro-Wilk test and expressed as mean ± standard deviation (SD) for normally distributed data or median (interquartile range) otherwise. Categorical variables were presented as frequencies and percentages. Comparison of *z*-scores was done using the independent samples t-test. Correlations between pituitary volume and clinical parameters were assessed using the Pearson correlation coefficient. 

## Results

Demographics and clinical characteristics

A total of 205 pediatric patients underwent MRI sella between January 2023 and June 2024. The cohort included 130 (63.4%) males and 75 (36.6%) females, with a mean age of 10.0 ± 3.37 years (range 2-17 years). Table 1 summarizes the demographic, auxological, and biochemical characteristics of the total cohort and subgroups.

**Table 1:** Demographic, auxological, and laboratory characteristics. Data are presented as mean (SD), except for the last three rows, which are presented as median (range). GHD, growth hormone deficiency; CPP, central precocious puberty; IGF-1, Insulin-like growth factor 1; IGFBP3, insulin-like growth factor-binding protein 3

Characteristic	Full population	GHD	CPP
Age (years)	10.04 (3.37)	10.02 (3.08)	8.02 (2.56)
Pituitary volume *z*-score	-1.46 (2.66)	-2.45 (1.6)	1.77 (4.11)
Height (cm)	128.7 (20)	126.79 (16.38)	136.19 (14.76)
Height *z*-score	-1.81 (1.56)	-2.15 (1.24)	0.62 (1.73)
Weight *z*-score	-1.53 (1.77)	-1.69 (1.32)	0.32 (1.78)
BMI *z*-score	-1.46 (2.66)	-0.67 (1.46)	0.2 (1.36)
Mid-parental height *z*-score	-0.84 (0.76)	-0.88 (0.76)	-0.8 (0.61)
Bone age (years)	9.61 (3.28)	9.02 (3.19)	9.6 (3.04)
Bone age delay (years)	-0.85 (1.64)	-1.27 (1.36)	1.3 (2.21)
Bone age *z*-score	-0.66 (1.57)	-1 (1.12)	1.4 (2.63)
IGF1 for age *z*-score, median (range)	-1.29 (-2.77 to 6.69)	-1.35 ( -2.14 to 2.21)	0.2 (-1.67 to 2.58)
IGF1 for Tanner *z*-score, median (range)	-1.18 (-3.74 to 5.4)	-1.23 (-2.95 to 5)	0.46 ( -2.09 to 5.4)
IGFBP3 *z*-score, median (range)	-1.03 (-4.04 to 3.21)	-1.12 ( -3.29 to 1.23)	0.7 (-3.72 to 3.21)

The mean height *z*-score was −1.81 ± 1.56, indicating overall short stature in the study population. Children with confirmed GHD had significantly lower mean height *z*-scores (−2.15 ± 1.24) and greater bone-age delay compared to the non-GHD group. The mean pituitary volume *z*-score was −1.46 ± 2.66 for the entire cohort and −2.45 ± 1.60 among patients with GHD, confirming a volumetric reduction pattern consistent with endocrine deficiency. The mean mid-parental height *z*-score was −0.84 ± 0.76, reflecting growth failure beyond familial short stature.

MRI indications

As shown in Table 2, the most frequent indication for MRI was suspected GHD (165, 80%), followed by precocious puberty (PP, 17, 8.3%), hypopituitarism (9, 4.4%), secondary growth failure (9, 4.4%), hyperprolactinemia (3, 1.5%), disorders of sex development (1, 0.5%), and diabetes insipidus (1, 0.5%).

**Table 2:** MRI sella indications. GHD, growth hormone deficiency

Indication	Frequency (*n*)	Percentage (%)
Suspected GHD	165	80%
Precocious puberty	17	8.3%
Hypopituitarism	9	4.4%
Secondary growth failure	9	4.4%
Hyperprolactinemia	3	1.5%
Disorder of sex determination (DSD)	1	0.5%
Diabetes insipidus (DI)	1	0.5%
Total	205	100%

Endocrinological diagnoses

Final diagnoses are summarized in Table 3. GHD was confirmed in 116 (56.5%) patients, with idiopathic short stature in 36 (17.6%), PP in 17 (8.3%), hypopituitarism in 9 (4.4%), familial short stature in 8 (3.9%), and constitutional delay of growth and puberty in 5 (2.4%), comprising the remainder.

**Table 3:** Final endocrinological diagnosis.

Condition	Frequency (*n*)	Percentage (%)
Growth hormone deficiency (GHD)	116	56.5%
Precocious puberty	17	8.3%
Hypopituitarism	9	4.4%
Familial short stature (FSS)	8	3.9%
secondary growth failure	9	4.4%
Idiopathic short stature (ISS)	36	17.6%
Constitutional delay of growth and puberty (CDGP)	5	2.4%
Hyperprolactinemia	3	1.5%
Disorder of sex determination (DSD)	1	0.5%
Diabetes insipidus (DI)	1	0.5%
Total	205	100%

MRI findings

Pituitary morphometry and structural findings are presented in Table 4. Nearly half of all patients (103, 50.2%) had abnormal pituitary volume measurements. Hypoplasia was the predominant abnormality, observed in 85 (41.5%) patients, most frequently in children with confirmed GHD (63, 54.3%). In contrast, hyperplasia was seen in 8 patients (3.9%), primarily in the PP subgroup (5, 29.4%), consistent with physiological pubertal enlargement.

**Table 4:** Pituitary volume measurements. The data are represented as number (%). GHD, growth hormone deficiency; CPP, central precocious puberty

Pituitary volume	Full population	GHD	CPP
Empty Sella	2 (1%)	2 (1.7%)	0 (0%)
Hypoplasia	85 (41.5%)	63 (54.3%)	1 (5.9%)
Borderline hypoplasia	7 (3.4%)	2 (1.7%)	0 (0%)
Normal	102 (49.8%)	47 (40.5%)	10 (58.8%)
Borderline hyperplasia	1 (0.5%)	0 (0%)	1 (5.9%)
Hyperplasia	8 (3.9%)	1 (0.9%)	5 (30.4%)
Total	205 (100%)	116 (100%)	17 (100%)

**Figure 1:**
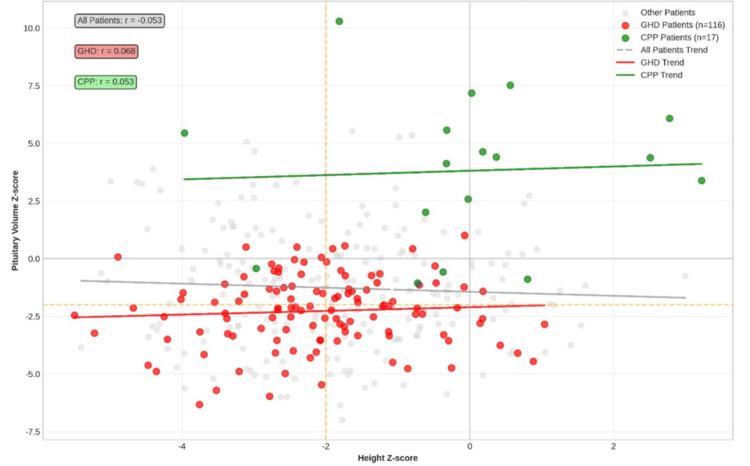
Correlation between diagnosis, height, and pituitary volume z-score. GHD, growth hormone deficiency; CPP, central precocious puberty

The mean pituitary volume *z*-score was significantly lower in the GHD group compared with the non-GHD group (−2.45 ± 1.60 vs. −0.84 ± 2.30; *P* < 0.01), confirming a strong association between pituitary volume reduction and biochemical GHD. Figure 1 demonstrates this correlation graphically across diagnostic categories.

Focal lesions

**Figure 2:**
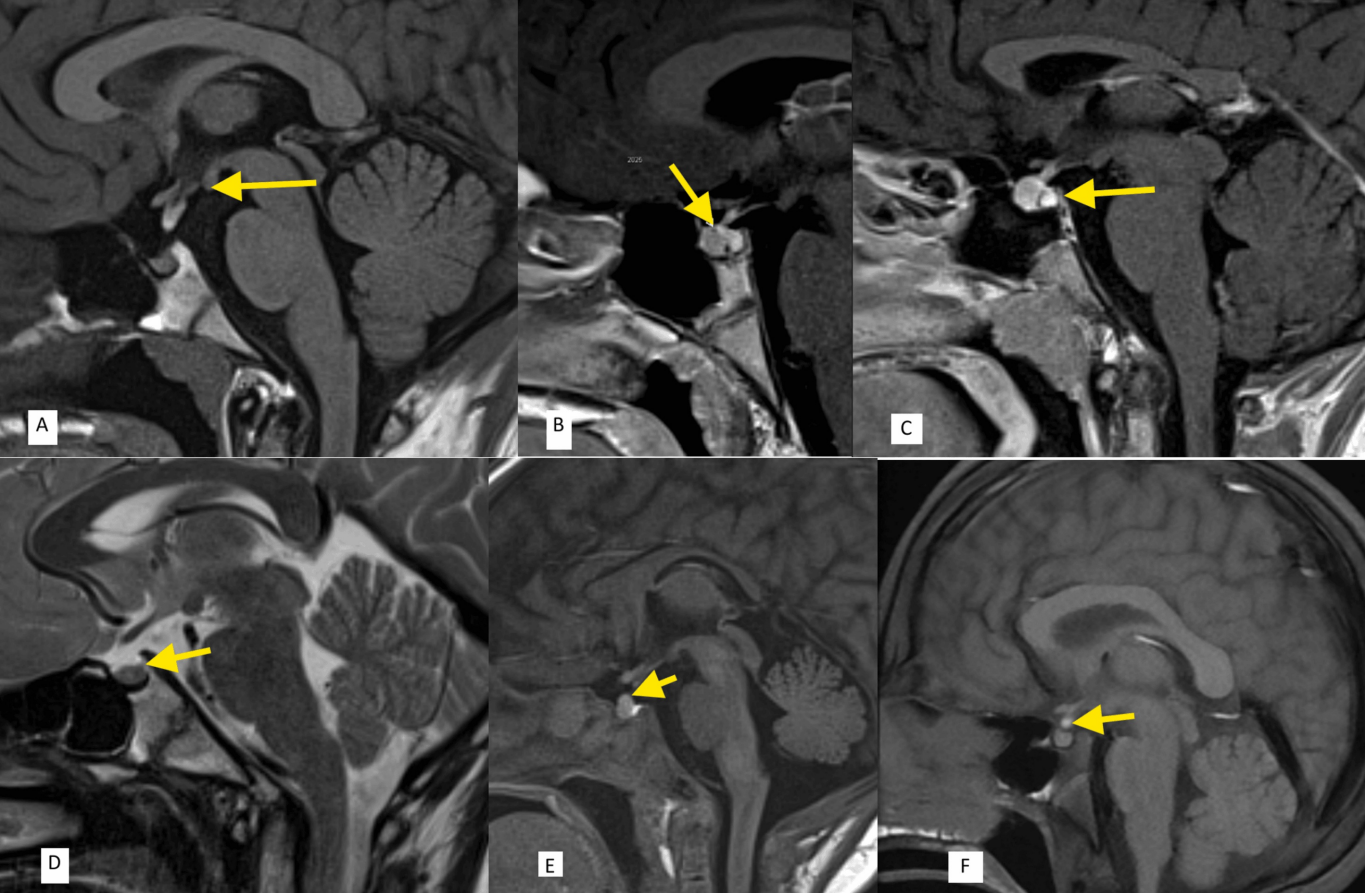
MRI images of pituitary lesions: (A) interrupted stalk syndrome (arrow); (B) microadenoma (arrow); (C) pituitary bulge (arrow); (D) Rathke's cleft cyst (arrow); (E) pituitary apoplexy (arrow); (F) ectopic posterior pituitary (arrow).

A total of 22 (10.7%) focal sellar or parasellar lesions were detected (Table 5). The most frequent lesion was Rathke’s cleft cyst (9, 4.4%), followed by interrupted pituitary stalk (5, 2.4%), microadenoma (3, 1.5%), ectopic posterior pituitary (1, 0.5%), and isolated Langerhans cell histiocytosis and pituitary apoplexy in one patient each (0.5%). MRI images of these lesions are presented in Figure 2.

**Table 5:** Pituitary lesions. The data are represented as number (%). GHD, growth hormone deficiency; CPP, central precocious puberty

Lesion	Full population	GHD	CPP
None	183 (89.3%)	106 (91.3%)	15 (88.2%)
Interrupted stalk	5 (2.4%)	3 (2.6%)	0 (0%)
Microadenoma	3 (1.5%)	2 (1.8%)	1 (5.9%)
Rathke’s cleft cyst	9 (4.4%)	4 (3.4%)	1 (5.9%)
Ectopic posterior pituitary	1 (0.5%)	1 (0.9%)	0 (0%)
Pituitary bulge	2 (1%)	0 (0%)	0 (0%)
Apoplexy	1 (0.5%)	0 (0%)	0 (0%)
Langerhans cell histiocytosis (LCH)	1 (0.5%)	0 (0%)	0 (0%)
Total	205 (100%)	116 (100%)	17 (100)

Notably, 10 (45%) lesions were found among patients with confirmed GHD, suggesting a predominance of structural abnormalities within this subgroup.

No cases of macroadenoma or mass effect were identified, and none required surgical or urgent endocrine intervention. These findings indicate that, in a tertiary pediatric cohort, the diagnostic yield of MRI sella is highest for structural hypoplasia in GHD and lowest for incidental lesions in PP.

## Discussion

This study describes MRI sella findings among children undergoing endocrine evaluation, particularly those assessed for GHD. In our cohort, more than half of children evaluated for suspected GHD were ultimately diagnosed with the condition, and structural abnormalities-most notably pituitary hypoplasia-were identified in 63 (54%) of confirmed GHD cases. This aligns with international literature reporting hypoplasia rates of 45%-60% among children with confirmed GHD [8-10,14-16].

Maghnie et al. reported that pituitary hypoplasia, ectopic posterior pituitary, and pituitary stalk interruption are among the most characteristic MRI abnormalities in GHD [14], and these findings closely mirror our observations. Similarly, recent regional data from Alyahyawi showed a high prevalence of pituitary hypoplasia (53%) in Saudi children with GHD [15], supporting the reproducibility of these structural markers in Middle Eastern populations.

The CPP subgroup exhibited a different radiological pattern. Although 5 (30.4%) showed pituitary hyperplasia, these changes are known to reflect physiological pubertal enlargement rather than pathology [3,17]. Only two CPP patients (12%) had focal lesions, consistent with evidence that MRI yield is low in older, asymptomatic girls but higher in boys and girls <6 years [11-13,17]. European Society for Pediatric Endocrinology (ESPE) guidelines recommend MRI for all boys, all girls <6 years, and any child with neurological signs, while advocating selective imaging in older girls - a pattern supported by our findings [11].

Across the entire cohort, 103 (50.2%) patients had abnormal pituitary volume, and 22 (10.7%) had focal lesions, with Rathke’s cleft cysts being the most common. The predominance of structural lesions in the GHD subgroup (10, 45% of all lesions) underscores the role of MRI in confirming suspected pituitary-based etiologies. Several studies have demonstrated that structural abnormalities on MRI are more likely in severe GHD or in children with multiple pituitary hormone deficiencies [10,14,18].

MRI provides complementary information in pediatric endocrine evaluation. GH stimulation tests are known to be variable, operator-dependent, and affected by age, BMI, and pubertal status; therefore, structural abnormalities serve as an important confirmatory element [18,19]. In addition, pituitary volume has been shown to correlate with auxology and may predict severity and persistence of GHD [16,20].

This study is the first from the UAE to comprehensively describe MRI Sella indications, structural findings, and endocrinological outcomes in a pediatric cohort. The results contribute important regional data and support the guideline-consistent use of MRI in the evaluation of GHD and selective use in CPP.

Limitations

This study has several limitations inherent to its retrospective, single-center, observational design. The absence of a control group and lack of longitudinal outcome data preclude assessment of diagnostic accuracy, impact on clinical management, or causal relationships between MRI findings and endocrine outcomes. As a tertiary referral center, the study population may be subject to selection bias, which may limit generalizability to broader pediatric settings. Although MRI interpretation and pituitary measurements were performed using standardized protocols by an experienced pediatric radiologist, interobserver variability and measurement bias cannot be fully excluded. In addition, analyses were primarily descriptive and did not adjust for potential confounding variables. These limitations should be considered when interpreting the findings, which are intended to describe local imaging patterns rather than establish clinical utility.

## Conclusions

MRI sella provides detailed anatomical characterization of the pituitary gland in children undergoing endocrine evaluation and reveals a spectrum of structural findings, particularly among those with growth hormone deficiency. In this retrospective cohort, pituitary hypoplasia was more frequently observed in children with confirmed GHD, while a substantial proportion had normal pituitary morphology, underscoring the heterogeneity of imaging findings in this population. In PP, imaging yield is lower and should follow guideline-based criteria emphasizing selective use in high-risk patients. Our findings contribute regional data on pituitary MRI utilization and outcomes in a tertiary pediatric endocrine setting and may inform future hypothesis-driven studies designed to evaluate the clinical impact and appropriate use of pituitary MRI.

## References

[1] Alatzoglou KS, Gregory LC, Dattani MT (2020). Development of the pituitary gland. Compr Physiol.

[2] Rey RA, Bergadá I, Ballerini MG (2024). Diagnosing and treating anterior pituitary hormone deficiency in pediatric patients. Rev Endocr Metab Disord.

[3] Çolaklar A, Fitoz ÖS (2023). Pituitary gland volumes in children with normal endocrine function. Pediatr Radiol.

[4] Sari S, Sari E, Akgun V (2014). Measures of pituitary gland and stalk: from neonate to adolescence. J Pediatr Endocrinol Metab.

[5] Go JL, Rajamohan AG (2017). Imaging of the sella and parasellar region. Radiol Clin North Am.

[6] Jipa A, Jain V (2021). Imaging of the sellar and parasellar regions. Clin Imaging.

[7] Fofanova O, Takamura N, Kinoshita E (2000). MR imaging of the pituitary gland in children and young adults with congenital combined pituitary hormone deficiency associated with PROP1 mutations. AJR Am J Roentgenol.

[8] Grimberg A, DiVall SA, Polychronakos C (2016). Guidelines for growth hormone and insulin-like growth factor-I treatment in children and adolescents: growth hormone deficiency, idiopathic short stature, and primary insulin-like growth factor-I deficiency. Horm Res Paediatr.

[9] (2000). Consensus guidelines for the diagnosis and treatment of growth hormone (GH) deficiency in childhood and adolescence: summary statement of the GH Research Society. GH Research Society. J Clin Endocrinol Metab.

[10] Bozzola M, Adamsbaum C, Biscaldi I (1996). Role of magnetic resonance imaging in the diagnosis and prognosis of growth hormone deficiency. Clin Endocrinol (Oxf).

[11] Carel JC, Léger J (2008). Clinical practice. Precocious puberty. N Engl J Med.

[12] Kaplowitz P, Bloch C (2016). Evaluation and referral of children with signs of early puberty. Pediatrics.

[13] Merke DP, Cutler GB Jr (1996). Evaluation and management of precocious puberty. Arch Dis Child.

[14] Maghnie M, Rossi A, di Iorgi N, Gastaldi R, Tortori-Donati P, Lorini R (2006). Hypothalamic-pituitary magnetic resonance imaging in growth hormone deficiency. Expert Rev Endocrinol Metab.

[15] Alyahyawi NY (2024). Auxological, clinical, and MRI abnormalities in pediatric patients with isolated growth hormone deficiency. Cureus.

[16] Abernethy L (1998). Imaging of the pituitary in children with growth disorders. Eur J Radiol.

[17] Oh YR, Kim YJ, Oh KE (2023). Brain magnetic resonance imaging (MRI) findings in central precocious puberty patients: is routine MRI necessary for newly diagnosed patients?. Ann Pediatr Endocrinol Metab.

[18] Alba P, Tsai S, Mitre N (2020). The severity of growth hormone deficiency does not predict the presence or absence of brain magnetic resonance imaging abnormalities: a retrospective review. Eur Endocrinol.

[19] Stanley T (2012). Diagnosis of growth hormone deficiency in childhood. Curr Opin Endocrinol Diabetes Obes.

[20] Li G, Shao P, Sun X, Wang Q, Zhang L (2010). Magnetic resonance imaging and pituitary function in children with panhypopituitarism. Horm Res Paediatr.

